# Supplementary material for: PCBs, PCNs, and PCDD/Fs in Soil around an Industrial Park in Northwest China: Levels, Source Apportionment, and Human Health Risk
Source: Int J Environ Res Public Health. 2023 Feb 16;20(4):3478. doi: 10.3390/ijerph20043478 (PMC9962420; doi:10.3390/ijerph20043478)
Supplement: Supplementary file 1 [file ijerph-20-03478-s001.zip › ijerph-2018332-supplementary.pdf]

Supporting Information

Text S1 Chemicals

Table S1 Native and internal standards for PCDD/Fs, PCNs and PCBs

Table S2 The concentrations of PCNs, PCDD/Fs, and PCBs in the soil samples (pg/g)

Fig. S1 Percentage composition of PCBs, PCNs, PCDDs, and PCDFs by mass (a) and TEQ (b) concentrations in soil samples collected around the industrial park

Fig. S2 Correlations between the proportion of PCN homologs in soil samples, flue gas samples from five converter steel plants and a cement kiln, seven commercial PCN formulations, and a commercial PCB formulation

Text S2 Positive matrix factorization model

Text S3 Equations of CR and no-CR assessment

Table S3 The parameters used in risk assessment

Table S4 Values of related parameters of 2,3,7,8-TCDD

Table S5 The human carcinogenic (CR) and non carcinogenic risks (no-CR) caused by PCBs, PCNs and PCDD/Fs in soil around the industrial park

Text S1 Chemicals

N-hexane and methylene chloride (pesticide grade) were purchased from J.T. Baker (Phillipsburg, USA). Silica gel (100–200 mesh) were purchased from Merck (Darmstadt, Germany). A PCDD/Fs standard (1613 STOCK) and PCDD/Fs internal standard (DF-LCS-C) were purchased from Wellington Laboratories (Guelph, Canada). PCBs standard (World Health Organization Congener Mix, indicator PCBs and CB-209) and PCBs internal standard were purchased from Cambridge Isotope Laboratories (Andover, MA). PCNs native standard (CN-2, 6/12, 13, 28/43, 27/30, 52/60, 66/67, 73, and 75) and internal standard (ECN-5102) were purchased from Wellington Laboratories and Cambridge Isotope Laboratories. Details of native and labeled standards can be found in Table S1.

Table S1 Native and internal standards for PCDD/Fs, PCNs and PCBs.

PCDD/Fs		***PCBs***		***PCNs***	
native standards	*internal standards*	*native standards*	*internal standards*	*native standards*	*internal standards*
2378-TCDF	$^{13}C_{12}$-2378-TCDF	CB28	$^{13}C_{12}$-CB28	CN2	$^{13}C_{10}$-CN42
12378-PeCDF	$^{13}C_{12}$-12378-PeCDF	CB52	$^{13}C_{12}$-CB52	CN6	$^{13}C_{10}$-CN42
23478-PeCDF	$^{13}C_{12}$-23478-PeCDF	CB77	$^{13}C_{12}$-CB77	CN13	$^{13}C_{10}$-CN42
123478-HxCDF	$^{13}C_{12}$-123478-HxCDF	CB81	$^{13}C_{12}$-CB81	CN28	$^{13}C_{10}$-CN42
123678-HxCDF	$^{13}C_{12}$-123678-HxCDF	CB101	$^{13}C_{12}$-CB101	CN27	$^{13}C_{10}$-CN27
234678-HxCDF	$^{13}C_{12}$-234678-HxCDF	CB105	$^{13}C_{12}$-CB105	CN52	$^{13}C_{10}$-CN52
123789-HxCDF	$^{13}C_{12}$-123789-HxCDF	CB114	$^{13}C_{12}$-CB114	CN66/67	$^{13}C_{10}$-CN67
1234678-HpCDF	$^{13}C_{12}$-1234678-HpCDF	CB118	$^{13}C_{12}$-CB118	CN68	$^{13}C_{10}$-CN67
1234789-HpCDF	$^{13}C_{12}$-1234789-HpCDF	CB123	$^{13}C_{12}$-CB123	CN73	$^{13}C_{10}$-CN73
OCDF	$^{13}C_{12}$- OCDF	CB126	$^{13}C_{12}$-CB126	CN75	$^{13}C_{10}$-CN75
2378-TCDD	$^{13}C_{12}$-2378-TCDD	CB156	$^{13}C_{12}$-CB156		
12378-PeCDD	$^{13}C_{12}$-12378-PeCDD	CB157	$^{13}C_{12}$-CB157		
123478-HxCDD	$^{13}C_{12}$-123478-HxCDD	CB138	$^{13}C_{12}$-CB167		
123678-HxCDD	$^{13}C_{12}$-123678-HxCDD	CB153	$^{13}C_{12}$-CB167		
123789-HxCDD	$^{13}C_{12}$-123789-HxCDD	CB167	$^{13}C_{12}$-CB167		
1234678-HpCDD	$^{13}C_{12}$-1234678-HpCDD	CB169	$^{13}C_{12}$-CB169		
OCDD	$^{13}C_{12}$-OCDD	CB180	$^{13}C_{12}$-CB180		
		CB189	$^{13}C_{12}$-CB189		
		CB209	$^{13}C_{12}$-CB189		

Table S2 The concentrations of PCNs, PCDD/Fs, and PCBs in the soil samples (pg/g)

Congener	P1	P2	P3	P4	P5	P6	P7	P8	P9	P10	P11	P12	P13
CN-2	53.21	12.11	30.01	28.06	33.76	15.47	19.27	98.71	14.69	35.11	13.12	9.77	24.08
CN-1	29.06	6.49	22.85	15.07	50.41	15.05	13.99	52.26	16.47	40.99	10.16	9.47	22.79
CN-4	14.95	7.87	54.86	15.40	9.31	39.23	28.05	28.10	48.54	64.83	11.88	28.42	72.71
CN-5/7	24.44	15.06	131.47	35.05	94.59	85.50	63.65	39.80	116.47	160.09	32.85	70.47	158.17
CN-6/12	7.24	1.79	12.32	5.68	13.91	8.44	10.17	13.84	10.99	17.64	4.13	6.03	13.55
CN-11/8	19.74	3.25	13.65	8.35	17.42	8.65	9.79	37.58	11.60	16.71	5.98	6.50	15.25
CN-3	19.99	9.42	53.25	18.10	12.19	35.56	33.52	35.21	48.05	59.77	12.96	25.12	63.44
CN-10	12.00	10.93	44.79	16.95	8.36	32.08	37.15	21.47	43.00	50.21	12.58	23.19	60.06
CN-9	2.94	0.52	2.30	1.25	8.11	1.38	1.66	4.33	2.28	3.24	1.21	1.03	2.43
CN-20	4.05	2.56	5.88	5.02	2.44	1.52	11.54	6.31	2.03	7.45	3.70	0.88	2.39
CN-19	20.95	0.92	1.81	9.03	2.34	1.39	3.99	30.48	1.79	2.85	2.01	1.09	2.39
CN-21	3.60	0.55	n.d.	1.66	0.91	n.d.	1.81	5.59	n.d.	n.d.	0.91	n.d.	n.d.
CN-24/14	25.57	21.09	102.04	35.69	21.46	76.69	98.08	41.42	110.39	134.91	35.40	60.96	158.16
CN-15	5.59	2.83	9.88	4.66	1.95	7.12	13.19	8.45	10.32	13.21	3.49	5.73	14.26
CN-16	5.67	0.56	2.41	1.81	1.07	1.49	2.64	8.26	1.82	3.05	1.17	1.37	2.19
CN-17/25/26	7.63	4.35	10.80	5.34	2.99	7.74	18.45	13.31	11.87	13.66	5.04	6.60	16.11
CN-13	12.75	12.91	35.56	12.82	8.09	24.62	48.03	21.67	36.09	41.10	12.94	18.77	50.42
CN-22	5.61	0.01	1.63	2.46	0.72	0.22	n.d.	8.59	0.33	2.23	0.66	0.31	0.74
CN-23	3.70	3.64	11.88	6.44	4.39	8.18	18.11	7.42	13.21	13.90	6.29	6.75	16.95
CN-18	1.11	0.41	0.83	0.65	0.53	0.63	2.98	1.52	1.23	1.01	0.78	0.74	1.24
CN-42	0.96	0.17	1.49	3.08	0.77	0.70	2.79	1.46	1.62	2.14	1.26	1.01	1.49
CN-37/33/34	5.66	1.48	4.68	7.71	2.74	3.51	5.52	7.33	7.22	6.93	4.87	4.00	6.54
CN-44/47	3.60	1.01	2.61	2.86	1.83	1.74	1.54	4.85	3.58	3.47	2.83	2.18	3.89
CN-45/36	n.d.	n.d.	n.d.	n.d.	n.d.	n.d.	n.d.	n.d.	n.d.	n.d.	n.d.	n.d.	n.d.
CN-28/43	45.70	3.60	15.00	17.25	5.30	3.66	12.95	57.48	6.13	20.02	5.49	3.74	8.65
CN-29	2.05	0.16	0.86	0.98	0.36	0.14	n.d.	2.70	0.46	0.59	0.49	0.36	0.42
CN-27/30	6.81	3.52	7.49	5.70	2.15	4.68	10.56	8.10	7.77	7.98	4.08	4.55	11.21
CN-39	4.29	n.d.	1.25	1.20	0.52	n.d.	n.d.	5.96	0.88	1.43	1.15	0.86	1.46
CN-32	0.31	n.d.	0.27	0.77	n.d.	0.08	n.d.	n.d.	0.67	0.46	0.21	0.31	0.62

CN-48/35	0.02	n.d.	0.84	1.16	0.43	0.42	0.01	0.03	1.88	1.23	1.03	1.01	1.77
CN-38/40	n.d.	0.19	2.70	1.67	2.06	2.48	5.17	0.11	6.75	4.42	4.68	3.61	6.18
CN-46	0.10	0.01	1.29	0.39	0.52	0.82	0.14	0.01	1.82	1.44	1.22	1.16	1.93
CN-31	n.d.	n.d.	0.22	0.06	n.d.	0.05	n.d.	n.d.	0.10	0.16	0.02	0.13	0.17
CN-41	0.67	n.d.	0.23	0.48	0.20	0.13	3.40	0.79	0.29	0.38	0.44	0.12	0.31
CN-52/60	6.69	1.71	3.44	11.48	3.28	2.40	11.04	6.25	5.69	4.88	5.88	3.08	4.09
CN-58	n.d.	0.11	2.76	4.90	0.58	0.81	2.84	0.90	2.08	3.05	1.10	1.30	1.97
CN-61	n.d.	n.d.	1.92	5.26	1.00	1.09	2.76	n.d.	5.56	2.57	2.38	2.78	2.74
CN-50	4.33	1.27	0.46	11.79	1.75	n.d.	4.56	3.10	4.95	5.89	2.63	3.56	3.69
CN-51	2.58	0.40	n.d.	1.66	1.50	n.d.	2.64	3.55	2.59	5.44	1.55	1.78	1.96
CN-54	2.80	0.77	1.13	1.97	0.95	0.80	1.85	2.82	1.48	2.74	0.91	0.92	1.00
CN-57	0.59	0.01	0.79	2.16	0.54	1.36	2.09	0.72	3.12	1.97	1.60	2.16	1.66
CN-62	0.01	n.d.	0.36	0.17	0.52	2.18	2.31	0.11	6.60	4.00	2.60	3.39	4.68
CN-53/55	n.d.	n.d.	0.15	n.d.	n.d.	0.46	n.d.	n.d.	2.50	0.97	1.59	2.22	3.06
CN-59	n.d.	n.d.	0.55	3.22	0.40	0.64	1.35	n.d.	4.76	1.65	1.55	1.87	2.27
CN-49	0.88	0.27	0.98	2.92	0.19	0.18	0.90	0.77	0.18	0.82	0.35	0.52	n.d.
CN-56	n.d.	n.d.	0.35	0.15	0.17	0.28	0.44	n.d.	0.56	0.88	0.21	0.58	0.06
CN-66/67	12.83	2.27	2.71	22.32	6.01	3.53	14.58	10.66	7.91	8.36	11.10	4.97	8.09
CN-64/68	0.05	0.72	1.53	19.86	2.08	1.63	n.d.	1.94	3.84	3.22	4.56	1.78	3.48
CN-69	0.01	0.26	0.54	0.16	1.68	1.53	0.06	1.14	3.82	2.68	3.93	1.86	3.15
CN-71/72	0.02	0.03	0.33	0.28	1.11	0.57	0.95	0.08	2.09	1.74	2.58	1.12	1.94
CN-63	1.25	0.49	0.76	6.44	0.95	0.29	2.71	1.08	1.76	1.71	2.05	1.22	1.43
CN-65/70	0.03	0.07	0.21	0.10	0.24	0.19	n.d.	0.03	0.40	0.41	0.60	0.27	0.57
CN-73	22.11	1.96	6.90	91.76	11.10	5.89	32.43	14.31	14.78	16.51	26.43	9.85	16.19
CN-74	3.49	n.d.	2.41	19.44	2.83	1.21	12.27	0.44	4.32	4.43	6.55	2.97	4.50
CN-75	68.93	3.16	27.49	222.57	14.58	7.10	227.40	37.38	18.51	25.56	40.16	10.61	17.72
2378-TCDD	n.d.	n.d.	n.d.	0.02	0.01	0.01	0.01	0.04	n.d.	n.d.	0.03	n.d.	n.d.
12378-PeCDD	0.26	0.02	0.02	0.08	0.10	0.04	0.04	0.16	0.11	0.04	0.05	0.02	0.01
123789-HxCDD	0.03	n.d.	0.06	0.06	0.18	0.01	0.03	0.18	0.04	0.07	0.02	0.04	0.01
123678-HxCDD	0.22	0.04	0.03	0.17	0.18	0.04	0.09	0.16	0.08	0.05	0.03	0.03	n.d.
123478-HxCDD	0.21	0.01	0.07	0.07	0.08	0.02	0.06	0.56	0.01	0.04	0.05	0.02	0.09
1234678-HpCDD	1.55	0.08	0.62	0.96	0.72	0.34	0.92	2.19	0.66	0.93	0.59	0.09	0.66
OCDD	3.62	0.56	2.18	2.03	3.14	1.86	1.88	34.45	1.44	3.06	1.89	0.62	7.72
2378-TCDF	0.65	0.05	n.d.	0.21	0.83	0.02	n.d.	n.d.	0.27	n.d.	0.61	n.d.	0.07
23478-PeCDF	1.04	0.24	0.03	1.02	0.46	0.04	0.40	0.78	0.49	0.72	0.30	0.11	0.16

12378-PeCDF	1.15	0.16	0.22	1.14	0.46	0.33	0.66	0.13	0.45	0.13	0.68	0.20	0.41
123478-HxCDF	1.27	0.13	0.43	2.95	0.83	0.38	0.89	1.29	0.18	0.86	0.99	0.16	0.53
123678-HxCDF	1.12	0.20	0.26	1.73	0.48	0.26	0.60	0.87	0.09	0.35	0.47	n.d.	0.25
123789-HxCDF	0.20	0.04	0.23	0.86	0.18	0.05	0.19	0.32	0.23	n.d.	0.21	0.08	0.17
234678-HxCDF	0.93	0.12	0.30	1.79	0.40	0.29	0.49	0.62	0.23	0.33	0.45	0.14	0.16
1234678-HpCDF	4.45	0.67	2.25	22.15	3.72	2.48	4.33	4.83	4.82	3.54	5.36	1.97	3.63
1234789-HpCDF	0.36	0.09	0.26	2.06	0.38	0.47	0.55	0.45	0.93	0.52	0.94	0.33	0.62
OCDF	5.80	1.19	11.29	118.45	29.25	29.63	50.09	11.98	60.96	29.80	76.07	30.21	42.60
CB-77	5.54	0.54	2.59	1.33	2.10	1.63	2.20	4.10	1.47	3.77	1.30	0.71	1.63
CB-81	0.62	0.13	0.24	0.03	0.31	0.24	0.41	0.63	0.15	0.46	0.08	0.26	0.24
CB-105	6.45	0.05	2.06	1.06	1.39	1.45	2.00	1.60	1.20	3.38	1.23	0.79	1.45
CB-114	2.56	0.13	0.40	0.54	0.77	0.31	0.41	0.05	0.40	0.67	0.33	0.04	0.31
CB-118	9.00	0.64	4.36	2.11	3.45	2.87	3.50	6.26	2.36	4.55	2.51	1.42	2.87
CB-123	n.d.	n.d.	0.05	n.d.	n.d.	0.06	n.d.	n.d.	0.06	n.d.	0.37	0.05	0.06
CB-126	2.58	0.13	0.13	n.d.	1.09	0.44	0.10	0.02	0.25	n.d.	0.42	0.29	0.44
CB-156	1.81	0.12	0.99	0.09	0.69	0.72	0.79	1.36	0.96	1.13	0.61	0.17	0.72
CB-157	0.47	0.06	0.10	0.27	0.38	0.03	0.34	0.55	0.10	0.32	0.35	0.15	0.03
CB-167	0.39	0.08	0.32	0.06	0.01	0.36	0.02	0.63	0.50	0.64	0.35	0.11	0.36
CB-169	0.45	0.12	0.30	0.47	0.26	0.07	0.37	0.45	0.27	0.12	0.10	0.06	0.07
CB-189	0.05	n.d.	0.13	0.02	0.36	0.35	0.13	0.12	0.25	0.33	0.28	0.00	0.35
CB-209	n.d.	2.13	195.10	302.81	396.27	545.61	616.94	0.00	1118.81	644.11	928.82	554.34	545.61
CB-28	65.40	5.45	85.14	24.49	15.05	86.84	23.45	40.96	92.09	109.41	19.55	46.23	86.84
CB-52	14.70	1.33	13.36	5.77	4.80	14.97	4.87	12.64	15.20	18.02	4.85	7.53	14.97
CB-101	8.18	0.33	3.51	3.79	1.93	4.11	1.73	5.22	3.51	4.47	2.22	1.39	4.11
CB-138	8.94	0.75	4.97	1.99	2.88	4.68	2.71	5.05	2.57	5.71	2.76	1.16	4.68
CB-153	8.94	0.75	3.68	2.14	3.39	4.85	4.59	5.15	3.20	6.21	2.73	1.63	4.85
CB-180	3.34	0.51	1.30	1.85	1.53	1.28	4.34	2.84	1.38	2.19	1.43	1.01	1.28

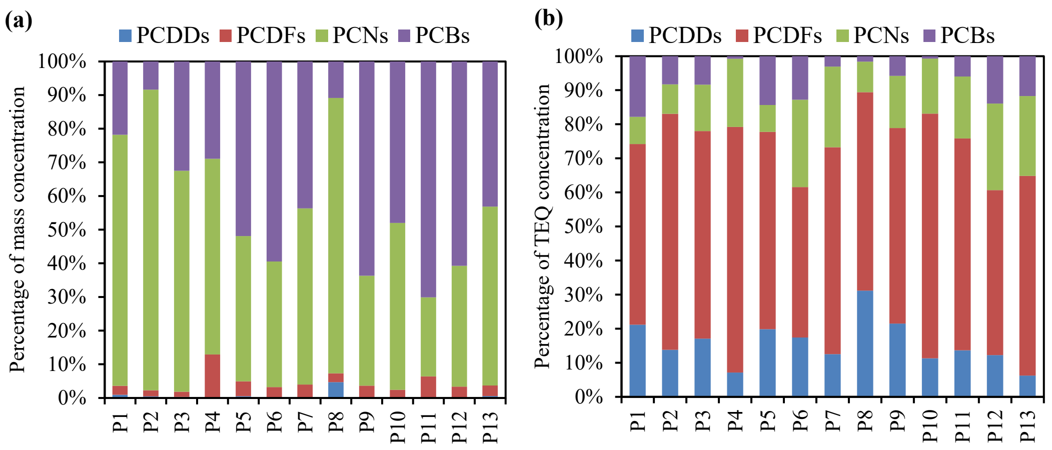

Fig. S1 Percentage composition of PCBs, PCNs, PCDDs, and PCDFs by mass (a) and TEQ (b) concentrations in soil samples collected around the industrial park.

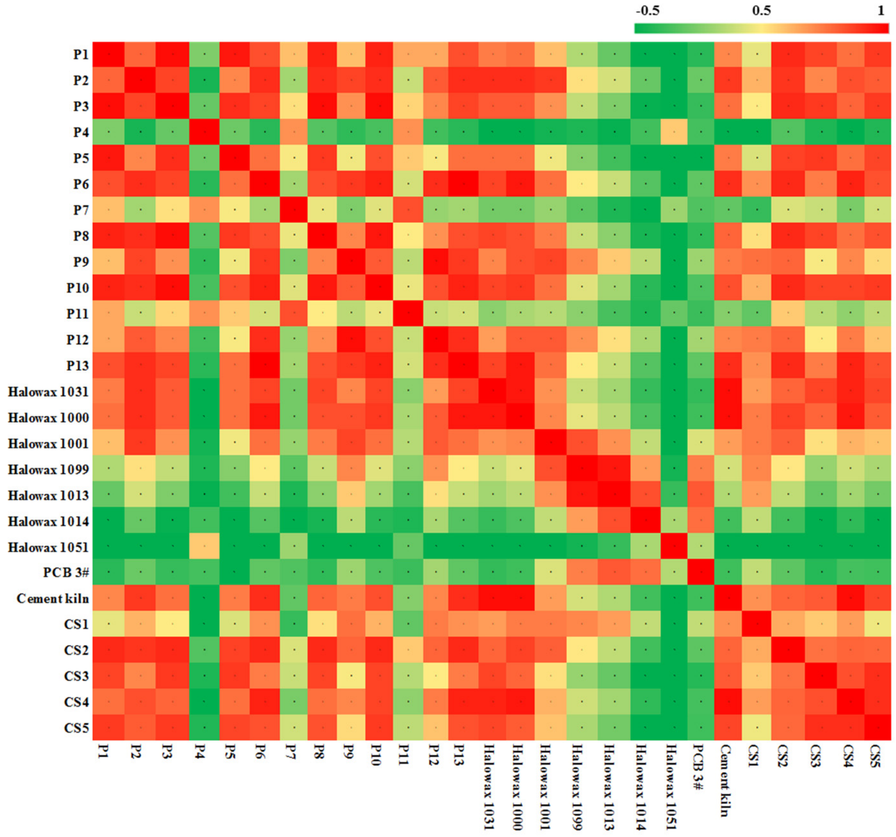

Fig. S2 Correlations between the proportion of PCN homologs in soil samples (P1–P13), flue gas samples from five converter steel plants (CS1–5) and a cement kiln, seven commercial PCN formulations (Halowax 1000,1001, 1013, 1014, 1031, 1051, and 1099), and a commercial PCB formulation (PCB 3#) (Li et al., 2014 [51]; Hu et al., 2013 [52]; Noma et al., 2004 [19]; Huang et al., 2015 [47]).

Text S2 Positive matrix factorization model

Positive matrix factorization model (PMF) was developed by the United States Environmental Protection Agency, and it can be used to identify the possible sources of POPs. Two files need to be put into PMF: (1) the concentration of each congener in samples and (2) the uncertainties of each congener in samples. For congeners whose concentration was lower than or equal to the detection limit, replaced their concentrations with one-half of the method detection limit (MDL) because negative values and zero are not permitted in the input files of PMF (Saba and Su, 2013 [62]; US EPA, 2014 [15]). The uncertainties (Unc) of them was calculated using a fixed fraction of the MDL (Equation S1) (US EPA, 2014 [15]):

$$\text{Unc} = \frac{5}{6} \times MLD \quad \text{(S1)}$$

For congeners whose concentration was greater than the MDL, the initial uncertainties were based on the following equation and the Error fraction was set as 10% as it used in USEPA model run examples and preview study (Saba and Su, 2013 [62]; US EPA, 2014 [15]):

$$\text{Unc} = \sqrt{(Error\ Fraction\ \times concentrarion)^2 + (0.5 \times MDL)^2} \quad \text{(S2)}$$

The uncertainty of each congener would be revised according to the S/N ratio before PMF run, which indicates whether the variability in MPF calculation within the noise or not (US EPA, 2014 [15]). Categorized the conger as "Weak" if S/N $\leq$ 1, and the uncertainty for that congener categorized "Weak" updated to three times the original uncertainty (US EPA, 2014 [15]).

As an exploratory analysis to determine the number of Factors, PMF simulations for 2, 3, 4 and 5 Factors were conducted. Q (robust) and Q (true) values are two important parameter used to determine the number of factors. The Q (true) is the goodness-of-fit parameter calculated including all points, and the Q (robust) is the goodness-of-fit parameter calculated excluding points not fit by the model (US EPA, 2014 [15]), which indicated the optimal goodness-of-fit at 4 Factors. Therefore, the 4 Factor model was chosen for simulations.

The PMF simulations of PCNs was conducted using 20 iterations and a random seed generator (default PMF parameters). Some of outputs from the PMF are showed below.

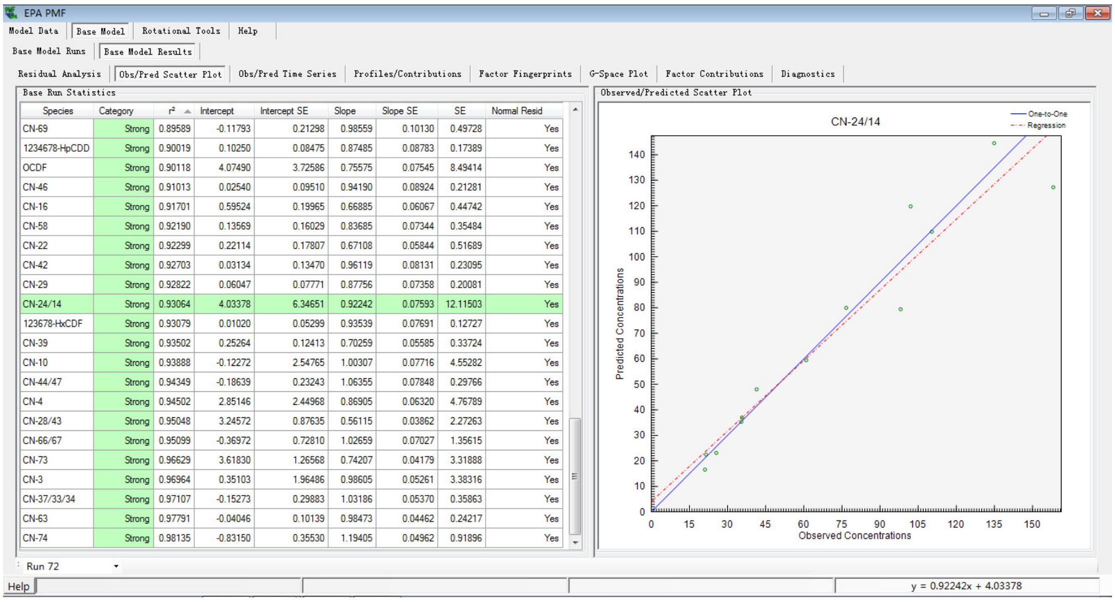

Species	Category	r²	Intercept	Intercept SE	Slope	Slope SE	SE	Normal Resid
CN-69	Strong	0.89589	-0.11793	0.21298	0.98559	0.10130	0.49728	Yes
1234678-HpCDD	Strong	0.90019	0.10250	0.08475	0.87485	0.08783	0.17389	Yes
OCDF	Strong	0.90118	4.07490	3.72586	0.75575	0.07545	8.49414	Yes
CN-46	Strong	0.91013	0.02540	0.09510	0.94190	0.08924	0.21281	Yes
CN-16	Strong	0.91701	0.59524	0.19965	0.66885	0.06067	0.44742	Yes
CN-58	Strong	0.92190	0.13569	0.16029	0.83685	0.07344	0.35484	Yes
CN-22	Strong	0.92299	0.22114	0.17807	0.67108	0.05844	0.51689	Yes
CN-42	Strong	0.92703	0.03134	0.13470	0.96119	0.08131	0.23095	Yes
CN-29	Strong	0.92822	0.06047	0.07771	0.87756	0.07358	0.20081	Yes
CN-24/14	Strong	0.93064	4.03378	6.34651	0.92242	0.07593	12.11503	Yes
123678-HxCDF	Strong	0.93079	0.01020	0.05299	0.93539	0.07691	0.12727	Yes
CN-39	Strong	0.93502	0.25264	0.12413	0.70259	0.05585	0.33724	Yes
CN-10	Strong	0.93888	-0.12272	2.54765	1.00307	0.07716	4.55282	Yes
CN-44/47	Strong	0.94349	-0.18639	0.23243	1.06355	0.07848	0.29766	Yes
CN-4	Strong	0.94502	2.85146	2.44968	0.86905	0.06320	4.76789	Yes
CN-28/43	Strong	0.95048	3.24572	0.87635	0.56115	0.03862	2.27263	Yes
CN-66/67	Strong	0.95099	-0.36972	0.72810	1.02659	0.07027	1.35615	Yes
CN-73	Strong	0.96629	3.61830	1.26568	0.74207	0.04179	3.31888	Yes
CN-3	Strong	0.96964	0.35103	1.96486	0.98605	0.05261	3.38316	Yes
CN-37/33/34	Strong	0.97107	-0.15273	0.29883	1.03186	0.05370	0.35863	Yes
CN-63	Strong	0.97791	-0.04046	0.10139	0.98473	0.04462	0.24217	Yes
CN-74	Strong	0.98135	-0.83150	0.35530	1.19405	0.04962	0.91896	Yes

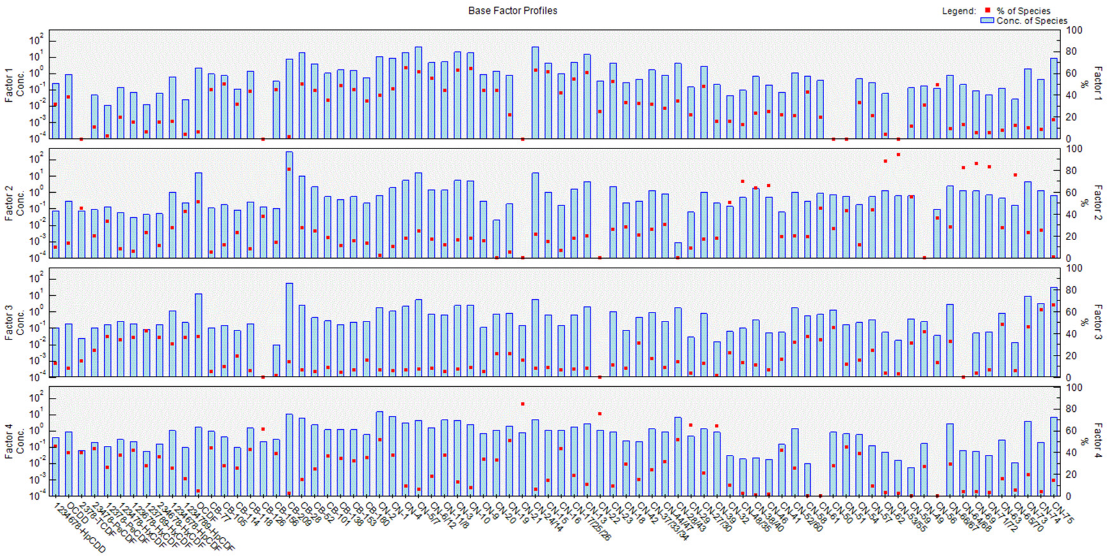

Text S2 Equations of CR and no-CR assessment

Carcinogenic risk:

$$CR_{der} = \frac{C_S \times DFS \times ABS \times 10^{-6}}{AT} \times \frac{SF_o}{GIABS} \quad (S3)$$

$$DFS = \frac{EF \times ED_C \times SA_C \times AF_C}{BW_c} + \frac{EF \times (ED_a - ED_c) \times SA_a \times AF_a}{BW_a} \quad (S4)$$

$$CR_{ing} = \frac{C_s \times SF_O \times RBA \times IFS \times 10^{-6}}{AT} \quad (S5)$$

$$IFS = \frac{EF \times ED_C \times IRS_C}{BW_c} + \frac{EF \times (ED_a - ED_c) \times IRS_a}{BW_a} \quad (S6)$$

$$CR_{inh} = \frac{C_s \times EF \times ED \times ET \times IUR \times 1000\ \mu g/mg}{AT} \times \left(\frac{1}{VF} + \frac{1}{PEF}\right) \quad (S7)$$

$$CR = CR_{der} + CR_{ing} + CR_{inh} \quad (S8)$$

Non carcinogenic risk:

$$no - CR_{der} = \frac{C_s \times EF \times ED \times SA \times AF \times ABS \times 10^{-6}}{RfD_O \times AT \times BW \times GIABS} \quad (S9)$$

$$no - CR_{ing} = \frac{C_s \times EF \times ED \times RBA \times IRS \times 10^{-6}}{RfD_O \times AT \times BW} \quad (S10)$$

$$no - CR_{inh} = \frac{C_s \times EF \times ED \times ET}{RfC \times AT} \times \left(\frac{1}{VF} + \frac{1}{PEF}\right) \quad (S11)$$

$$no\text{-}CR = no\text{-}CR_{der} + no\text{-}CR_{ing} + no\text{-}CR_{inh} \quad (S12)$$

Where:

CR: Total carcinogenic risk

CR_{der}: Carcinogenic risk caused by skin contact of volatile substances and fugitive dust

CR_{ing}: Carcinogenic risk caused by ingestion of volatile substances and fugitive dust

CR_{inh}: Carcinogenic risk caused by inhalation of volatile substances and fugitive dust

no-CR: Total non carcinogenic risk

no-CR_{der}: Non carcinogenic risk caused by skin contact of volatile substances and fugitive

no-CR_{ing}: Non carcinogenic risk caused by ingestion of volatile substances and fugitive dust

no-CR_{inh}: Non carcinogenic risk caused by inhalation of volatile substances and fugitive dus

Table S3 The parameters used in risk assessment (USEPA, 2017 [17]; USEPA, 1991 [16])

Parameter		units	adult	
IFS	Soil ingestion factor	mg kg^{-1}	36750	
DFS	Soil dermal contact factor	mg kg^{-1}	103390	
AT_{-CR}	Averaging time	d	25550	
			children	adult
EF	Exposure freqency	d y^{-1}	350	350
ED	Exposure duration	y	6	26
ET	Exposure time	hh^{-1}	24/24	24/24
AT_{-no-CR}	Averaging time	d	2190	9490
BW	Body weight	kg	15	80
IRS	Soil ingestion rate	mg d^{-1}	200	100
SA	Surface area	cm^2 d^{-1}	2373	6032
AF	Adherence factor soil to skin	mg cm^2	0.2	0.07

Table S4 Values of related parameters of 2,3,7,8-TCDD (USEPA, 2017 [17])

Parameter		units	Values (2,3,7,8-TCDD)
RfDo	Non carcinogenic reference dose	mg $kg^{-1}d^{-1}$	7.00×10^{-10}
RfC	Non carcinogenic reference concentration	mg m^{-3}	4.00×10^{-8}
ABS	Dermal absorption from soil	-	0.03
VF	Volatilization coefficient	m^3 kg^{-1}	1.96×10^{6}
PEF	Fugacity coefficient of particles	m^3 kg^{-1}	1.36×10^{9}
Sfo	Carcinogenic slope factor	(mg kg^{-1} $d^{-1})^{-1}$	1.30×10^{5}
IUR	Unit inhalation risk factor	$(\mu g/m^3)^{-1}$	38
RBA	Relative bioavailability	–	1
GIABS	Gastrointestinal absorption rate	–	1

Table S5 The human carcinogenic (CR) and non carcinogenic risks (no-CR) caused by PCBs, PCNs and PCDD/Fs in soil around the industrial park

		**P1**	**P2**	**P3**	**P4**	**P5**	**P6**	**P7**	**P8**	**P9**	**P10**	**P11**	**P12**	**P13**
**CR**	PCDDs	0.0686	0.0058	0.0098	0.0286	0.0343	0.0134	0.0184	0.0685	0.0266	0.0142	0.0218	0.0058	0.0053
**(1.0×10-6)**	PCDFs	0.1718	0.0291	0.0350	0.2904	0.1001	0.0340	0.0892	0.1280	0.0710	0.0900	0.0992	0.0230	0.0494
	PCNs	0.0259	0.0036	0.0078	0.0806	0.0136	0.0197	0.0348	0.0198	0.0190	0.0202	0.0290	0.0121	0.0197
	PCBs	0.0578	0.0035	0.0048	0.0031	0.0248	0.0099	0.0045	0.0035	0.0072	0.0009	0.0096	0.0066	0.0099
	Total	0.3241	0.0421	0.0574	0.4027	0.1729	0.0770	0.1469	0.2197	0.1238	0.1253	0.1596	0.0475	0.0842
**no-CR**	PCDDs	0.0063	0.0005	0.0009	0.0026	0.0032	0.0012	0.0017	0.0063	0.0025	0.0013	0.0020	0.0005	0.0005
	PCDFs	0.0158	0.0027	0.0032	0.0267	0.0092	0.0031	0.0082	0.0118	0.0065	0.0083	0.0091	0.0021	0.0045
	PCNs	0.0024	0.0003	0.0007	0.0074	0.0013	0.0018	0.0032	0.0018	0.0017	0.0019	0.0027	0.0011	0.0018
	PCBs	0.0053	0.0003	0.0004	0.0003	0.0023	0.0009	0.0004	0.0003	0.0007	0.0001	0.0009	0.0006	0.0009
	Total	0.0298	0.0039	0.0053	0.0371	0.0159	0.0071	0.0135	0.0202	0.0114	0.0115	0.0147	0.0044	0.0078
